# Risk factors for *Plasmodium falciparum* infection in pregnant women in Burkina Faso: a community-based cross-sectional survey

**DOI:** 10.1186/s12936-021-03896-8

**Published:** 2021-09-06

**Authors:** Jean Baptiste Yaro, Alphonse Ouedraogo, Amidou Diarra, Salif Sombié, Z. Amidou Ouedraogo, Issa Nébié, Chris Drakeley, Sodiomon B. Sirima, Alfred B. Tiono, Steven W. Lindsay, Anne L. Wilson

**Affiliations:** 1 Centre National de Recherche et de Formation sur le Paludisme, Ouagadougou, Burkina Faso; 2 Department of Biosciences, Durham University, Durham, UK; 3 Groupe de Recherche et d’Action en Santé, Ouagadougou, Burkina Faso; 4 London School of Hygiene and Tropical Medicine, London, UK; 5 Department of Vector Biology, Liverpool School of Tropical Medicine, Liverpool, UK

## Abstract

**Background:**

Malaria in pregnancy remains a public health problem in sub-Saharan Africa. Identifying risk factors for malaria in pregnancy could assist in developing interventions to reduce the risk of malaria in Burkina Faso and other countries in the region.

**Methods:**

Two cross-sectional surveys were carried out to measure *Plasmodium falciparum* infection using microscopy in pregnant women in Saponé Health District, central Burkina Faso. Data were collected on individual, household and environmental variables and their association with *P. falciparum* infection assessed using multivariable analysis.

**Results:**

A total of 356 pregnant women were enrolled in the surveys, 174 during the dry season and 182 during the wet season. The mean number of doses of sulfadoxine–pyrimethamine for Intermittent Preventive Treatment in pregnancy (IPTp-SP) was 0.4 doses during the first trimester, 1.1 doses at the second and 2.3 doses at the third. Overall prevalence of *P. falciparum* infection by microscopy was 15.7%; 17.8% in the dry season and 13.7% in the wet season. 88.2% of pregnant women reported sleeping under an insecticide-treated net (ITN) on the previous night. The odds of *P. falciparum* infection was 65% lower in women who reported using an ITN compared to those that did not use an ITN (Odds ratio, OR = 0.35, 95% CI 0.14–0.86, p = 0.02). IPTp-SP was also associated with reduced *P. falciparum* infection, with each additional dose of IPTp-SP reducing the odds of infection by 44% (OR = 0.56, 95% CI 0.39–0.79, p = 0.001). Literate women had a 2.54 times higher odds of *P. falciparum* infection compared to illiterate women (95% CI 1.31–4.91, p = 0.006).

**Conclusions:**

The prevalence of *P. falciparum* infection among pregnant women remains high in Burkina Faso, although use of IPTp-SP and ITNs were found to reduce the odds of infection. Despite this, compliance with IPTp-SP remains far from that recommended by the National Malaria Control Programme and World Health Organization. Behaviour change communication should be strengthened to encourage compliance with protective malaria control tools during pregnancy.

**Supplementary Information:**

The online version contains supplementary material available at 10.1186/s12936-021-03896-8.

## Background

Malaria in pregnancy is a major public health problem in sub-Saharan Africa. The World Health Organization (WHO) estimates that 33.2 million women in Africa were at risk of malaria in pregnancy in 2019, with 35% of these (11.6 million) infected with malaria, resulting in 822,000 infants born with low birth weight [1]. Pregnant women are more susceptible to malaria and its adverse effects than non-pregnant women and infection with *Plasmodium falciparum* can lead to poor outcomes for the mother, the fetus and child [2]. Pregnant women are also more attractive to *Anopheles gambiae*, the most important African malaria vector, due to increased release of carbon dioxide and attractive volatiles, and hotter bodies [3, 4]. Pregnant women infected with *P. falciparum* can develop placental malaria, with sequestration of the parasite in the placental vasculature [5]. This can lead to maternal anaemia and death, pre-term delivery, stillbirth, low birth weight, with long-term impacts on child growth and cognitive development. In areas of stable transmission, primigravid women are at greatest risk of malaria infection and have higher parasite densities [6]. Over successive pregnancies there appears to be a boosting of immunity to *P. falciparum* that reduces parasite density and prevents disease [6]. Prevention and management of malaria in pregnancy is three-pronged: the use of insecticide-treated nets [ITNs, distributed free-of-charge at Antenatal Clinic (ANC) visits], intermittent preventive treatment in pregnancy (IPTp) with sulfadoxine–pyrimethamine (SP) and prompt access to diagnosis and effective case management [7].

Burkina Faso is a high burden country and despite high coverage of ITNs and prompt and effective treatment with anti-malarials is not experiencing declines in malaria [8]. Malaria transmission intensity is extremely high with children aged 5–15 years in south-west Burkina Faso receiving on average 3 infective bites per week during the 6 month transmission season [9]. Additional reasons for stagnating progress in malaria control may also include insecticide resistance [10, 11], outdoor biting of malaria vectors [12, 13], insufficient coverage and use of ITNs, or rapid loss of ITNs due to holing [14]. Burkina Faso has a high burden of malaria in pregnancy with one study conducted in a rural health district in south-west Burkina Faso in 2014 reporting an incidence of 39.2 per 1000 women-months, with the burden of infection in primigravids more than twice as high as that in multigravids at 88.6 per 1000 women-months [15]. In 2014, another study in Bobo-Dioulasso city in south-west Burkina Faso, identified 18.1% of pregnant women had *P. falciparum* infection [16]. According to national guidelines in Burkina Faso, pregnant women are advised to receive at least three doses of IPTp-SP starting from the second trimester, with a minimum interval of 1 month between doses [17]. IPTp-SP is administered at ANC visits by facility-based health workers and is provided free of charge, along with ITNs.

There have been many studies of risk factors for malaria in pregnancy in sub-Saharan Africa, where increased risk was reported to be associated with younger age in pregnancy, primigravidae, first trimester of pregnancy, non-use of ITNs, lack of education and HIV co-infection [15, 16, 18, 19]. Few, however, have evaluated socioeconomic and environmental risk factors for malaria in pregnancy. For example, recently a number of studies have shown that malaria in children is associated with poor housing [9, 20, 21], but it is not known whether this is also true for pregnant women. The goal of the present study was to identify risk factors for *P. falciparum* infection in pregnancy in Saponé Health District, central Burkina Faso, including potential socioeconomic and environmental risk factors, during the wet season and dry season. Identifying risk factors for malaria in pregnancy could assist in developing interventions to reduce malaria burden in pregnancy in Burkina Faso and other countries in sub-Saharan Africa.

## Methods

### Study design

Risk factors for *P. falciparum* infection were measured during two cross-sectional surveys, one at the beginning of the dry season in December 2018 and the second one at the end of the rainy season from September to October 2019.

### Study site

**Fig. 1:**
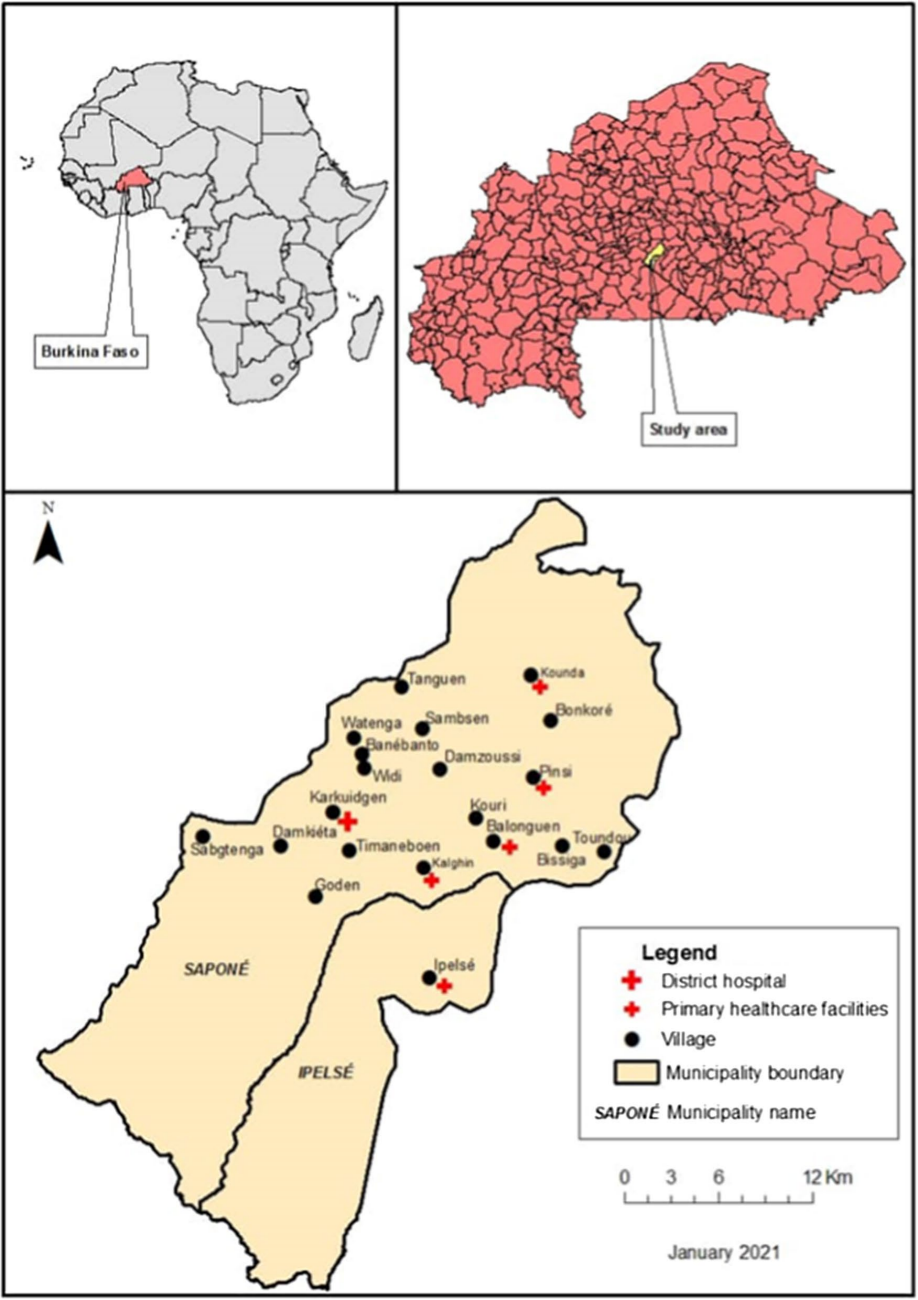
Map of the study site

The study was conducted within the Saponé Health and Demographic Surveillance system (HDSS), situated in the central region of Burkina Faso, 45 km south-west of Ouagadougou city, the capital of Burkina Faso (Fig. 1). The Saponé HDSS covers 1600 km^2^, with a total estimated population of 102,000 living in 83 villages and 10,841 compounds [22, 23]. Compounds are georeferenced and a census of the population is conducted annually. Malaria transmission is intense and highly seasonal [24], with the peak of malaria transmission occurring at the end of the rainy season (June to October) and markedly reduced transmission during the dry season (December to May) [25]. The main vectors are *An. gambiae sensu stricto*, *Anopheles arabiensis* and *Anopheles funestus*, and *P. falciparum* accounts for > 95% of all malaria infections [24–26]. This is a rural area of open Sudanian savannah, where farming is dominant and the major crops grown are sorghum and millet. Houses in the study area are typically constructed with mud walls and floors, with thatched or metal roofs [27]. The population is served by 23 local health facilities and a district hospital.

### Screening and enrolment

The HDSS census listings were not up-to-date and so study personnel worked closely with Community Health Workers who identified women of child-bearing age in the study villages. Home visits were conducted in 20 villages in the HDSS. The 20 villages were selected purposively because of their close proximity to Ouagadougou and their accessibility. Villages closest to Ouagadougou along the axis of the National Road 7 were selected until the sample size was reached. Enrolment at home was adopted, rather than screening at the ANC because ANC attendance is relatively low in the study area, with only 35% of women attending the ANC at least three times [28]. All study procedures were carried out at the home of the study participant. Women thought to be pregnant were invited to take a dipstick pregnancy test, which was performed by fieldworkers at the woman’s home. Women identified as pregnant, but who had not yet visited their ANC, were reminded to attend their local health facility. The study protocol and procedures were explained by trained staff to potential participants in French or the main local language of Moore.

### Inclusion and exclusion criteria

Pregnant women were enrolled if they were between 15 and 40 years, provided written informed consent and agreed with the study procedures, including taking of blood. Women found to have clinical malaria were eligible for enrolment in the study. Pregnant women with a known history of SP allergy or any other medical condition that in the opinion of a study physician may be a threat to her or the fetus were not recruited into the study. Women who were pregnant at both surveys were eligible for enrolment in the study, but no women were enrolled where this was the case.

### Parasitological assessment

Each participant provided a finger prick blood sample (100 µL) for malaria infection detection and characterization. *Plasmodium falciparum* quantitative sexual and asexual parasite count and qualitative species identification was performed by microscopy. Two blood smears were prepared and read by two independent experienced microscopists based at the Centre National de Recherche et de Formation sur le Paludisme (CNRFP) according to established standard operating procedures. Discrepancies in positive and negative reads and parasite counts differing by more than ten-fold between the two reads were resolved by the supervisor. In case of fever (axillary temperature ≥ 37.5 °C or reported fever in last 24 h) or other symptoms/signs of clinical malaria, a rapid diagnostic test for malaria (SD BIOLINE Malaria Ag P.f/Pan, Abbott Laboratories, Illinois, USA) was performed. Subjects presenting with clinical malaria were referred to the nearest health facility and treated according to national guidelines [17].

### Risk factor data collection

All study participants completed a questionnaire (Additional file 1), where demographic data, medical and obstetric history, including previous ANC visits, IPTp-SP doses and use of anti-malarials within 14 days prior to study enrolment were recorded. ANC attendance and IPTp-SP doses were transcribed from the participants health card. Fieldworkers recorded information about the household, including whether the woman had access to an ITN and whether she slept under an ITN the previous night. Women sleeping under an ITN were asked about the bed net source and how many times they left their ITN during the previous night. ITN fabric integrity was also assessed by fieldworker observation and classified as entire/complete, with any hole, or torn. Women were asked to estimate the time they went to bed and the time they get out of bed in the morning. Social and economic risk factors for malaria were recorded, including ethnicity, education level and occupation, ownership of a radio or mobile phone, estimated distance to the nearest health facility, and use of other protective measures, including mosquito coils, insecticide sprays, traditional spatial repellent or commercial topical repellents.

House construction (metal or thatched roof, presence of open eaves, electricity supply to sleeping room), household size (number of persons) and the presence of clothes hanging in the sleeping room were recorded. The presence or absence of large domestic animals (donkey, horses, sheep, cows, goats, dogs) and rubbish within 5 m of each study participant’s household was also recorded.

### Sample size

Assuming a 20% prevalence of *P. falciparum* infection in pregnant women [16], a sample size of 346 women provided greater than 80% power to detect effect sizes of > 50% (odds ratio = 2) at the 5% level of significance, given a 50% prevalence of the risk factor of interest in the population. The sample size was calculated using the formula for calculation of sample size for un-matched cross-sectional studies (www.openepi.com/SampleSize/SSCohort.htm). In determining the effect size we considered housing type since improved housing has been found to reduce malaria prevalence by ~ 50% [29].

### Data management and statistical analysis

Data were collected on Android personal digital assistants programmed using Open Data Kit (https://getodk.org/) and included drop down boxes and consistency checks to reduce data entry errors. Following cleaning, the dataset was locked and saved in Microsoft Access and analysed with Stata 15 (Statacorp, Texas, USA).

Descriptive analysis was performed including calculation of percentages for categorial variables and mean and standard deviation for continuous variables. Geometric mean was calculated for asexual stage parasite density (geometric mean parasite density, GMPD). Categorical variables were compared using a Chi-squared test, and normally distributed continuous variables using a t-test. The primary outcome measure was the prevalence of microscopically confirmed *P. falciparum* infection in pregnant women during each cross-sectional survey. Logistic regression was used to investigate the association between independent variables (risk factors) and the primary outcome (dependent variable), adjusting for clustering by village. The wet and dry season surveys were analysed together with season evaluated as an independent risk factor. A multivariable model was constructed using a forwards stepwise process and models compared using a Wald test. Variables were tested in the multivariable model if p ≤ 0.05 in the univariable model. Correlations between variables were tested using a Pearson’s coefficient (r) and variables that were correlated with r ≥ 0.5 or ≤ − 0.5 were omitted from the multivariable model. Interactions between independent variables were evaluated. Odds ratios and adjusted odd ratios with 95% confidence intervals were computed and Wald test p-values presented. P-values were not corrected for multiple comparisons.

## Results

### Socio-demographic characteristics of pregnant women

A total of 356 pregnant women were enrolled in the surveys, 182 during the wet season and 174 during the dry season (Table 1). The mean age of the study participants was 26.9 years, ranging from 15 to 40 years old, and was similar in both surveys. Of these women, 78 (21.9%) were in their first pregnancy, 74 (20.8%) in their second and 204 (57.3%) in their third pregnancy or more. Women aged under 20 years were primarily primigravidae (32/34, 94.1%), while women aged 30–40 were primarily multigravidae (125/131, 95.4%). Most women were enrolled in their second (132/276, 47.8%) and third trimester of pregnancy (108/276, 39.1%) where gestational age was recorded (gestational age was missing for 80 women). Fewer women were enrolled at their first trimester compared to other trimesters; only 6.4% (5/78) of primigravidae, 14.9% (11/74) of secundigravidae and 9.8% (20/204) of multigravidae were in their first trimester. 59.0% (210/356) of women were illiterate and most were farmers (249/356, 69.9%) or traders (79/356, 22.1%). 73.1% (57/78) of primigravidae were literate compared to only 42.5% of those on their second pregnancy (31/73) and 27.6% of women with two or more pregnancies (56/203). 97.5% of study participants were from the Mossi ethnic group (347/356). Most women lived in households with three or fewer people 57.9% (206/356). Only 46.3% (165/356) of women reported having an electricity supply in the sleeping room. Most houses were constructed with metal roofs (340/356, 95.5%) with 64.6% (230/356) of houses having clothes hanging in the sleeping room. Large domestic animals were common near the house (281/356, 78.9%), and 45.8% (163/356) of participants reported solid waste within 5 m of their households. Characteristics of the women surveyed in the dry and wet season tended to be similar, although in the wet season the distance to the nearest health centre was longer (40.2% > 3 km) than in the dry season (46.1% > 3 km), and in the dry season there were more likely to be clothes hanging in the sleeping room (80.5%) than in the wet season (49.5%).

**Table 1:** Characteristics of the study participants and households

Variables	Dry season, n (%)	Wet season, n (%)	Total n (%)
	N = 174	N = 182	N = 356
Age (years)			
< 20	11 (6.3)	23 (12.6)	34 (9.6)
20–30	101 (58.1)	90 (49.5)	191 (53.7)
30–40	62 (35.6)	69 (37.9)	131 (36.8)
Education			
Illiterate	107 (61.5)	103 (56.6)	210 (59.0)
Literate	65 (37.4)	79 (43.4)	144 (40.4)
Occupation			
Farmers	115 (66.1)	134 (73.6)	249 (69.9)
Traders	46 (26.4)	33 (18.1)	79 (22.1)
Other	11 (6.3)	12 (6.6)	23 (6.5)
Gravidity			
Primigravida	31 (17.8)	47 (25.8)	78 (21.9)
Secundigravida	42 (24.1)	32 (17.6)	74 (20.8)
Multigravida	101 (58.1)	103 (56.6)	204 (57.3)
Gestation^a^			
1st trimester	19 (10.9)	17 (9.3)	36 (10.1)
2nd trimester	75 (43.1)	57 (31.3)	132 (37.1)
3rd trimester	61 (35.1)	47 (25.8)	108 (30.3)
Ethnic group			
Mossi	169 (97.1)	178 (97.8)	347 (97.5)
Fulani	4 (2.3)	2 (1.1)	6 (1.7)
Other	1 (0.6)	2 (1.1)	3 (0.8)
Roof material of sleeping room			
Metal	165 (94.8)	175 (96.2)	340 (95.5)
Non-metal (Thatch/mud)	7 (4.0)	6 (3.3)	13 (3.7)
Eave status of sleeping room			
Closed	*	30 (16.5)	–
Open	*	149 (81.9)	–
Electricity supply in the sleeping room			
No	91 (52.3)	87 (47.8)	178 (50.0)
Yes	72 (41.4)	93 (51.1)	165 (46.3)
Presence of large domestic animals within 5 m of the household			
No	31 (17.8)	37 (20.3)	68 (19.1)
Yes	138 (79.3)	143 (78.6)	281 (78.9)
Presence of solid waste within 5 m of the household			
No	90 (51.7)	98 (53.8)	188 (52.8)
Yes	79 (45.4)	84 (46.2)	163 (45.8)
Household size			
1–3	92 (52.9)	114 (62.6)	206 (57.9)
4–5	68 (39.1)	55 (30.2)	123 (34.6)
≥ 6	8 (4.6)	13 (7.1)	21 (5.9)
Distance to health facility (km)			
< 3	100 (57.5)	98 (53.9)	198 (55.6)
3–5	42 (24.1)	65 (35.7)	107 (30.1)
> 5	28 (16.1%)	19 (10.4)	47 (13.2)
Hanging clothes in the sleeping room			
No	28 (16.1)	92 (50.5)	120 (33.7)
Yes	140 (80.5)	90 (49.5)	230 (64.6)

^a^Gestational age was missing for 80 women

*Eave status was accidentally omitted during dry season survey

### Parasitological characteristics of pregnant women

The overall prevalence of *P. falciparum* infection (asexual stage) by microscopy was 15.7% (56/356), with 17.8% (31/174) during the dry seasonal survey and 13.7% (25/182) in the wet season survey (p = 0.3) (Table 2). No significant difference was found in the prevalence of parasitaemia by gravidity (primigravid = 14/78, 17.9%, secundigravid = 12/74, 16.2%, multigravidae = 30/204, 14.7%, p = 0.8). 2.0% (7/356) of women were found to have clinical malaria and were referred to their local health facility for treatment.

**Table 2:** Parasitological characteristics, ANC attendance and use of personal protection according to season

Variables	Dry season, N = 174				Wet season, N = 182			
	Primigravidity	Secundigravidity	Multigravidity	Total	Primigravidity	Secundigravidity	Multigravidity	Total
	n = 31	n = 42	n = 101		n = 47	n = 32	n = 103	
Parasitological characteristics								
Parasitaemia (any level)^a^	7 (22.6%)	10 (23.8%)	14 (13.9%)	31 (17.8%)	7 (14.9%)	2 (6.3%)	16 (15.5%)	25 (13.7%)
Parasitaemia ≥ 1000/µl^a^	4 (12.9%)	2 (4.8%)	4 (4.0%)	10 (5.7%)	5 (10.6%)	1 (3.1%)	6 (5.8%)	12 (6.6%)
**GMPD/µl (95% CI)^a^	1435.7 (412.7–4995.2)	738.7 (276.0–1976.8)	478.9 (263.7–869.7)	705.7 (444.8–1119.5)	2925.3 (421.0–20,325.6)	Low number of observations	469.7 (160.3–1376.3)	876.2 (367.0–2092.2)
ANC attendance								
None	6 (19.4%)	14 (35.0%)	28 (28.0%)	48 (28.1%)	3 (6.4%)	7 (21.9%)	17 (16.5%)	27 (14.8%)
1	7 (22.6%)	11 (27.5%)	28 (28.0%)	46 (26.9%)	8 (17.0%)	7 (21.9%)	24 (23.3%)	39 (21.4%)
2	4 (12.9%)	3 (7.5%)	22 (22.0%)	29 (17.0%)	6 (12.8%)	7 (21.9%)	25 (24.3%)	38 (20.9%)
3	7 (22.6%)	7 (17.5%)	12 (12.0%)	26 (15.2%)	16 (34.0%)	5 (15.6%)	20 (19.4%)	41 (22.5%)
4 or more	7 (22.6%)	5 (12.5%)	10 (10.0%)	22 (12.9%)	14 (29.8%)	6 (18.8%)	17 (16.5%)	37 (20.3%)
Use of personal protective measures								
Access to ITN	23 (74.2%)	37 (88.1%)	93 (93.0%)	153 (88.4%)	41 (87.2%)	32 (100%)	99 (96.1%)	172 (94.5%)
Used an ITN the previous night	21 (67.7%)	35 (83.3%)	85 (85.0%)	141 (81.5%)	42 (89.4%)	31 (96.9%)	100 (97.1%)	173 (95.1%)
Mosquito coils	8 (25.8%)	9 (21.4%)	24 (24.0%)	41 (23.7%)	10 (21.3%)	3 (9.4%)	15 (14.6%)	28 (15.4%)
Other spatial repellent	1 (3.2%)	0	5 (5.0%)	6 (3.5%)	4 (8.5%)	1 (3.1%)	10 (9.7%)	15 (8.2%)
Commercial repellent (topical)	3 (9.7%)	5 (11.9%)	9 (9.0%)	17 (9.8%)	8 (17.0%)	1 (3.1%)	8 (7.8%)	17 (9.4%)
0 dose of IPTp-SP	6 (19.4%)	15 (35.7%)	16 (15.8%)	37 (21.3%)	0	4 (12.5%)	7 (6.8%)	11 (6.0%)
1 doses of IPTp-SP	9 (29.0%)	8 (19.0%)	35 (34.7%)	52 (29.9%)	11 (23.4%)	8 (25.0%)	28 (27.2%)	47 (25.8%)
2 doses of IPTp-SP	7 (22.6%)	4 (9.5%)	23 (22.8%)	34 (19.5%)	12 (25.5%)	9 (28.1%)	35 (34.0%)	56 (30.8%)
3 or more doses of IPTp-SP	5 (16.1%)	8 (19.0%)	12 (11.9%)	25 (14.4%)	20 (42.6%)	6 (18.8%)	21 (20.4%)	47 (25.8%)
Mean IPTp-SP dose (95% CI)	1.4 (1.0–1.8)	1.3 (0.8–1.7)	1.4 (1.2–1.6)	1.4 (1.2–1.6)	2.2 (1.9–2.5)	1.8 (1.3–2.3)	1.8 (1.6–2.1)	1.9 (1.8–2.1)
Use of anti-malarial drug 2 weeks before the survey	3 (10.7%)	6 (15.0%)	18 (18.4%)	27 (16.3%)	7 (14.9%)	2 (6.3%)	10 (9.7%)	19 (10.4%)

*ANC* antenatal clinic, *CI* confidence interval, *GMPD* geometric mean parasite density, *IPTp-SP* intermittent preventive treatment in pregnancy with sulfadoxine pyrimethamine, *ITN* insecticide-treated net, *SD* standard deviation

^a^Reported for asymptomatic infections and clinical malaria

The overall GMPD of infected pregnant women was 777.3/µl (95% CI = 496.0–1218.2). GMPD was slightly higher in the wet season [876.2/µl (95% CI = 367.0–2092.0)] than in the dry season [705.7/µl (95% CI = 444.8–1119.5)] but this result was not significant (p = 0.20). GMPD was higher in women in their first pregnancy (2049.4/µl, 95% CI = 753.5–5573.7) compared to those in their second pregnancy or more (562.6/µl, 95% CI = 347.3–911.5, p = 0.02). There was no difference in the proportion of women in each trimester by gravidity in the dry season (p = 0.5) or wet season (p = 0.3). GMPD was higher in women aged under 20 years old than older women, with a GMPD of 3374.7/µl (95% CI = 946.1–12036.9) among women aged under 20 years and 633.5/µl (95% CI = 368.0–1090.7) among women aged 20–30 and 552.0/µl (95% CI = 204.8–1487.5) among women aged 30 years or more. *P. falciparum* gametocyte carriage was rare (6/356, 1.7%).

### Use of preventive measures against malaria

ANC attendance was higher in the wet season than in the dry season, with 20.3% (37/182) of women attending four or more times in the wet season, compared to 12.9% (22/174) in the dry season (p = 0.01). In the wet season, a significantly higher proportion of primigravidae women had attended four or more ANC visits (14/47, 29.8%) compared to women who had two (6/32, 18.8%) or more pregnancies (17/103, 16.5% p = 0.02). Although a similar pattern was observed in the dry season, the differences were not significant (primigravid = 7/31, 22.6%, secundigravid = 5/42, 12.5%, multigravid = 10/101, 10.0%, p = 0.29).

At the time of the survey, women had received on average 1.7 doses of IPTp-SP (95% CI = 1.5–1.8) with increasing number of doses according to the trimester of pregnancy (0.4, 1.1 and 2.3 doses at first, second and third trimester of pregnancy, respectively). 19.4% (7/36) of women in the first trimester reported receiving IPTp-SP, despite this not being recommended until the second trimester. Primigravidae were more likely to report taking at least one IPTp-SP dose (72/78, 92.3%) than secundigravidae (55/74, 74.3%) or multigravidae (181/204, 88.7%) (p = 0.002). Women aged under 20 years (31/34, 91.2%) or women aged over 30 years (120/131, 91.6%) were more likely to report to report taking IPTp-SP than women aged 20–30 years (157/191, 82.2%) (p = 0.04). There was no difference in the proportion of literate and illiterate pregnant women reporting taking IPTp-SP in this study (p = 0.8).

A total of 95.1% (173/182) of women reported using an ITN in the rainy season survey, compared to 81.5% (141/174) in the dry season survey (p < 0.001). 95.2% (339/356) of women reported that the National Malaria Control Programme provided their ITNs. The mean age of the ITN was 7.9 months (standard deviation = 8.2) and 89.9% (320/356) of them were reported to be un-holed. On average women self-reported an estimated time to bed of 20.21 h during the dry season and 20.13 h during the rainy season, and left the bed at 05.29 h during the dry season and 5:39 h during the wet season. Only 4.5% (16/356) of pregnant women reported that they did not leave their ITN until the morning. However, 47.2% (168/356) of them exited their ITN once or twice a night, and 42.7% (152/356) exited their ITN three or more times a night. Mosquito coils were used by 19.4% (69/356) of participants, while 5.9% (21/356) used other types of spatial repellent (insecticide sprays or traditional repellents such as herbs) and 9.6% (34/356) used topical commercial repellents.

### Risk factors for *P. falciparum* infection

Univariable analysis indicated lower odds of *P. falciparum* infection among women who were in the 3rd trimester compared to the 1st trimester; aged 30–40 years compared to those aged less than 20 years; reported using ITNs; attended more ANC visits; and received more IPTp-SP doses. Univariable analysis indicated a higher prevalence of *P. falciparum* infection in literate women compared to illiterate women. Analysis of correlations between variables indicated that gestation was correlated with ANC attendance (r = 0.60) and number of IPTp-SP doses (r = 0.60), ANC attendance was correlated with number of IPTp-SP doses (r = 0.82) and gravidity was correlated with age (r = 0.59). Since women will receive additional IPTp-SP doses with increased ANC attendance and increased gestational age, we excluded gestation and ANC attendance from the variables considered. Based on this, reported ITN use, the number of IPTp-SP doses, education and age group were evaluated in the multivariable model. Multivariable analysis showed that the odds of *P. falciparum* infection in pregnancy was reduced among pregnant women who reported using ITNs (Odds ratio, OR = 0.35, 95% CI 0.14–0.86, p = 0.02) after adjusting for number of IPTp-SP doses, education and age group (Table 3). The odds of *P. falciparum* infection was also reduced with use of IPTp-SP, with each additional dose reducing the odds by 44% (OR = 0.56, 95% CI 0.39–0.79, p = 0.001) after adjusting for ITN use, education and age group. Literate women were at higher odds of *P. falciparum* infection than illiterate women (OR = 2.54, 95% CI 1.31–4.91, p = 0.006) after adjusting for ITN use, number of IPTp-SP doses and age group. The Wald test indicated that age group improved the overall model fit but age group was not significant after adjusting for reported ITN use, number of IPTp-SP doses and education (20–30 years compared to < 20 years OR = 0.77, 95% CI 0.25–2.37, p = 0.64, 30–30 years compared to < 20 years, OR = 0.76, 95% CI 0.31–1.84, p = 0.54).

**Table 3:** Risk factors for *P. falciparum* infection in pregnant women in Saponé Health District

Factors	*P. falciparum* infection positivity, n/N (%)	Univariable analysis			Multivariable analysis		
	N = 356	Odds ratio	95% CI	p-value	Odds ratio	95% CI	p-value
Pregnancy characteristics							
Gestation							
1st trimester	10/48 (20.8)	1					
2nd trimester	34/132 (25.8)	1.32	0.90–1.94	0.16			
3rd trimester	7/108 (6.5)	0.26	0.12–0.58	0.001			
Gravidity							
Primigravidae	14/78 (17.9)	1					
Secundigravidae	12/74 (16.2)	0.88	0.48–1.64	0.70			
Multigravidae	30/204 (14.7)	0.79	0.41–1.50	0.47			
Socio-demographic characteristics							
Mean age (years)	–	0.98	0.94–1.02	0.35			
Age group (years)							
< 20	8/34 (23.5)	1			1		
20–30	34/191 (17.8)	0.70	0.36–1.39	0.31	0.77^a^	0.25–2.37	0.64
30–40	14/131 (10.7)	0.39	0.22–0.69	0.001	0.76^a^	0.31–1.84	0.54
Education							
No formal education	24/210 (11.4)	1			1		
Literate	31/144 (21.5)	2.13	1.29–3.52	0.003	2.54^b^	1.31–4.91	0.006
Occupation							
Farmers	34/249 (13.7)	1					
Traders	15/79 (19.0)	1.48	0.75–2.93	0.26			
Other	6/23 (26.1)	2.23	0.72–6.90	0.16			
Use of personal protective measures							
ITN use the previous night							
No	12/41 (29.3)	1			1		
Yes	43/314 (13.7)	0.38	0.18–0.81	0.01	0.35^c^	0.14–0.86	0.02
Number of exits from the ITN the previous night							
2 or more times	18/152 (11.8)	1					
Less than 2 times	31/184 (16.8)	1.51	0.84–2.71	0.17			
ANC attendance (number of visits)		0.53	0.43–0.66	< 0.001			
Number of IPTp-SP doses (one unit increase)	–	0.57	0.41–0.80	0.001	0.56^d^	0.39–0.79	0.001
Mosquito coils							
No	46/286 (16.1)	1					
Yes	9 /69 (13.0)	0.78	0.48–1.29	0.33			
Other spatial repellent							
No	54/335 (16.1)	1					
Yes	2/21 (9.5)	0.55	0.09–3.53	0.53			
Commercial repellent (topical)							
No	51/320 (15.9)	1					
Yes	4/34 (11.8)	0.70	0.24–2.04	0.52			
Distance to nearest health centre (km)							
< 3	26/198 (13.1)	1					
3–5	19/107 (17.8)	1.43	0.73–2.78	0.30			
> 5	10/47 (21.3)	1.79	0.66–4.83	0.25			
Use of anti-malarial drug during the last 2 weeks before the survey							
No	48/302 (15.9)	1					
Yes	5/46 (10.9)	0.65	0.32–1.31	0.23			
House characteristics and construction							
Household size							
< 4	31/206 (15.0)	1					
4 ≤ no. < 6	20/123 (16.3)	1.10	0.58–2.07	0.78			
≥ 6	4/21 (19.0)	1.33	0.36–4.91	0.67			
Roof material of sleeping room							
Metal	52/340 (15.3)	1					
Thatch or mud	3/13 (23.1)	1.66	0.62–4.42	0.31			
Electricity supply in sleeping room							
No	33/178 (18.5)	1					
Yes	21/165 (12.7)	0.64	0.37–1.11	0.11			
Clothes hanging in sleeping room							
No	16/120 (13.3)	1					
Yes	38/230 (16.5)	1.29	0.58–2.84	0.53			
Asset ownership							
Own a radio							
No	24/127 (18.9)	1					
Yes	31/224 (13.8)	0.74	0.42–1.30	0.29			
Own a mobile phone							
No	8/56 (14.3)	1					
Yes	47/295 (15.9)	1.14	0.62–2.09	0.68			
Environmental factors							
Season enrolled							
Dry season	31/174 (17.8)	1					
Rainy season	25/182 (13.7)	0.73	0.41–1.31	0.30			
Presence of large domestic animals within 5 m of the household							
No	10/68 (14.7)	1					
Yes	45/281 (16.0)	1.11	0.48–2.55	0.81			
Presence of solid waste within 5 m of the household							
No	30/188 (16.0)	1					
Yes	24/163 (14.7)	0.91	0.50–1.64	0.75			

*CI* confidence interval, *IPTp-SP* intermittent preventive treatment in pregnancy with with sulphadoxine pyrimethamine, *ITN* insecticide-treated net

^a^Adjusted for reported ITN use, the number of IPTp-SP doses and education

^b^Adjusted for ITN use, number of IPTp-SP doses and age group

^c^Adjusted for number of IPTp-SP doses, education and age group

^d^Adjusted for ITN use, education and age group

## Discussion

This study aimed to identify risk factors for malaria infection in pregnant women living in an area of intense and stable seasonal malaria transmission in Burkina Faso with high levels of pyrethroid resistance in malaria vectors. The overall prevalence of *P. falciparum* infection during both surveys was 15.7% and is similar to that recorded in other studies in Burkina Faso (e.g. 18.1% [16]) and other high burden countries in sub-Saharan Africa, e.g. 20.1% in Kenya [30] and 21.6% in Ghana [31]. These results suggest that *P. falciparum* malaria infection is common in pregnant women in the community and that malaria burden in pregnancy remains high despite the use of standard malaria control interventions. The overall geometric mean of parasites density in the study area was 777.3/µl (95% CI 496.0–1218.2). Fana and co-workers from Nigeria, another high burden country, recorded a similar mean parasite density of 800/µl [19].

No difference in parasite prevalence by gravidity was observed, which differs from other studies, which found higher prevalence in primi- and secundigravidae, compared to multigravidae [32, 33]. This may be because in our study setting pregnant women are, irrespective of gravidity, exposed to same high level of transmission, and hence the probability of getting infected is quite similar. Parasite density was, however, higher in primigravidae and secundigravidae compared to multigravidae and in younger women compared to older women, since younger women are more likely to be primigravid. This may be because primigravid and secundigravid women are less immunologically competent to control parasite density compared to multigravidae. There was no significant difference in *P. falciparum* prevalence between the wet season (13.7%) and dry season (17.8%) or in GMPD between the two surveys. This may be because the dry season survey was conducted at the start of the dry season when infections from the end of the rains may still be present. *P. falciparum* gametocyte carriage was low in this study (1.7%). Low parasite density may result in lower gametocyte identification by microscopy.

The study found that IPTp-SP and ITNs are highly effective interventions for preventing malaria infection during pregnancy. For each additional dose of IPTp reported as being received by women, the odds of malaria infection fell by 44%. At the time of the survey, relatively few women, had however taken three or more doses of IPTp-SP (20.2%) which is recommended by the NMCP and WHO [1, 17]. Fewer women had received three or more doses of IPTp-SP in the dry season (14.4%), than in the wet season (25.8%), and ANC attendance was lower in the dry season. It may be that women perceive a lower risk of malaria transmission in the dry season due to less mosquito biting pressure.

ITNs were associated with 65% reduction in the odds of *P. falciparum* infection, which is higher than other studies have found [34]. This indicates that ITNs are protective against malaria in pregnancy in Burkina Faso despite high levels of insecticide resistance present in the country [10, 35]. This contrasts with findings from two studies conducted in south-west Burkina Faso; a cohort study in children aged 5–15 years in which showed no difference in malaria risk between ITN users and non-users [9], and a cross-sectional survey in all ages which found no difference in infection risk between ITN users and non-users (Yaro et al., unpublished). Overall, 91.3% of pregnant women owned an ITN, with 88.2% reporting using an ITN the night before the survey. This is similar to other surveys from Burkina Faso; in the Banfora Region, 80.6% of surveyed children reported sleeping under an ITN the previous night [9]. The high reported ITN use is encouraging, although accurately determining net use is challenging and reporting can be susceptible to response bias [36]. Women reported going to bed at 20.21 h during the dry season and 20.13 h during the rainy season. This finding contrasts with a study by Guglielmo and co-workers who reported that all females in south-west Burkina Faso (sample of 211 and 695 females observed in two villages) were outdoors until 22.00 h, after which point women started to move indoors to bed [12]. It may be that pregnant women tend to go to bed earlier and so those using ITNs are more likely to be protected from vector biting during the early evening which has been observed in Burkina Faso [12].

The finding that literate women had 2.54 times the odds of *P. falciparum* infection compared to illiterate women was unexpected. While literature on this is mixed, with some studies showing an association between literacy and reduced infection [32] and others showing no such association [33, 37], to the best of our knowledge there are no other studies which found a higher odds of infection in literate women. There was no association between literacy and ITN use, ANC attendance or IPTp-SP doses, suggesting that literacy is not acting via reduced uptake of preventive measures. Again here, existing literature is mixed with some studies finding an association between increased literacy and increased use of ITNs and IPTp [38, 39], others finding, as this study did, no such association, [40, 41] or an inverse association, including a study of malaria indicator surveys from eight African countries [42]. Literate women were more likely to be primigravid than illiterate women, however, this cannot explain the finding since gravidity was not associated with *P. falciparum* infection in the model. It may be that literate women have higher risk occupations or are spending more time outdoors, potentially subject to early evening vector biting [12]. This finding requires further assessment to understand the mechanism by which literacy can increase infection risk.

A strength of this study is that it adopted community-level recruitment of pregnant women. This was an unusual approach since most studies assess risk factors for *P. falciparum* infection among those women attending ANC. The study population was probably more representative of the true situation since it also included women that did not necessarily know they were pregnant or women who had not yet attended an ANC. The lack of sampling frame from which to select the women of childbearing age may have, however, introduced bias in the selection of the study participants. A limitation of the study is the sample size which may have been insufficient to identify minor risk factors such as house construction details which may have been less common in the study population than assumed or been protective to a smaller extent than the study was powered for. Study villages were selected purposively based on their accessibility to Ouagadougou which may have meant that the study participants were not representative of the Saponé HDSS, perhaps due to differences in socioeconomic status if living closer to the A7 road afforded better economic opportunities than the more isolated rural villages. While we did collect information on asset ownership, this did not allow us to determine a wealth index or socioeconomic position, which may have been an important determinant of *P. falciparum* infection. We did not collect information on knowledge, attitudes and practices in relation to malaria in pregnancy and use of protective measures. This is an area for future research and will be informative for improvement of delivery of existing interventions and for design of novel interventions. Multiple comparisons were made and, therefore, some of the associations noted may have occurred by chance alone.

What are the implications of this research for control of malaria in pregnancy in Burkina Faso? Behaviour change communication is necessary to ensure high ANC attendance and compliance with IPTp-SP and ITN use. Based on the study findings, messaging for ANC attendance in the study area should target primigravid women due to their higher GMPD, literate women due to apparent higher infection risk and encourage early attendance irrespective of season. As is common in sub-Saharan Africa, pregnant women are often unaware that they are pregnant and so do not attend or are unwilling to attend an ANC in the early stages of pregnancy. An association between early ANC attendance and a higher average number of IPTp-SP doses has been demonstrated in several studies [43–45]. One option to increase IPTp-SP coverage is community delivery by community health workers, rather than ANC. This delivery route has been shown in a clinical trial in Burkina Faso to increase IPTp-SP compliance from 2.1 to 2.8 doses in the community delivery study arm with no apparent decrease in ANC attendance [46]. Seven women reported receiving IPTp-SP despite being in the first trimester. IPTp-SP is not recommended in the first trimester due to concerns over congenital abnormalities [47]. If this is a true finding, then increased health worker sensitization is necessary. While ultrasound can determine gestational age with precision, it is often unaffordable or inaccessible for many women in Burkina Faso.

## Conclusion

The prevalence of *P. falciparum* infection among pregnant women was 15.7% despite wide deployment of ITNs and access to IPTp-SP. Nonetheless, the odds of *P. falciparum* infection was reduced by 44% for each additional IPTp-SP dose taken and women who reported using an ITN had a 65% lower odds of *P. falciparum* infection. These findings suggest that IPTp-SP and ITNs use are effective at reducing malaria infection in pregnant women living in malaria high burden countries, even where there is high insecticide resistance, but that research is needed to increase uptake of IPTp-SP. The finding of literate women having 2.54 times the odds of *P. falciparum* infection compared to illiterate women requires further research to elucidate the underlying mechanism.

## Supplementary Information

**Additional file 1.** Formulaire de selection enrolement.

## Data Availability

The datasets used and/or analysed during the current study are available from the corresponding author on reasonable request.

## Abbreviations

ANC: Antenatal clinic
CI: Confidence interval
CNRFP: Centre National de Recherche et de Formation sur le Paludisme
GMPD: Geometric mean parasite density
HDSS: Health and Demographic Surveillance system
HIV: Human immunodeficiency virus
IPTp: Intermittent Preventive Treatment in pregnancy
ITN: Insecticide-treated net
OR: Odds ratio
SD: Standard deviation
SP: Sulfadoxine pyrimethamine
WHO: World Health Organization
