# Supplementary material for: Risk factors for Plasmodium falciparum infection in pregnant women in Burkina Faso: a community-based cross-sectional survey
Source: Malar J. 2021 Sep 6;20:362. doi: 10.1186/s12936-021-03896-8 (PMC8422625; doi:10.1186/s12936-021-03896-8)
Supplement: Supplementary file 1 — Additional file 1. Formulaire de selection enrolement. [file 12936_2021_3896_MOESM1_ESM.pdf]

FORMULAIRE DE SELECTION ENROLEMENT

Site de l'Etude: ☐ **Saponé** ☐ **Kaya** **Village**: ……………………………………………
☐ **Femme enceinte** ☐ **Femme non enceinte** ☐ **Enfant**
Enquête Transversale: ☐ Saison pluvieuse ☐ Saison sèche

Date (DD/MMM/YYYY): |__|__|/|__|__|__|/|__|__|__|_ _|

I. Données Démographiques

Nom: ____

ID DSS: |__|__|__|__|__|__|__|__|__|__|__|__| **N° de Sélection:** |__|__|-|__|__|__|

Date de naissance: |__|__|/|__|__|__|/|__|__|__|__| or Age: |__|__| ans **Initiales:** |__|__|__|

II. Explication et signature du consentement

1. Le participant a t-il été informé au sujet de l'étude? ☐ Oui, ☐ Non
2. Le participant ou le RLA a t-il signé le formulaire de consentement? ☐ Oui, ☐ Non

   Si oui, completer la date de signature du consentement: |__|__|/|__|__|__|/|__|__|__|__|

   Si applicable, completer la date de signature de l'assentiment: |__|__|/|__|__|__|/|__|__|__|__|

III. Histoire médicale actuelle

1. Le participant a t-il a problème de santé connu? ☐ Yes, ☐ No

   Si oui, spécifier: ---

2. Existe t-il une fièvre ou histoire de fièvre des dernières 24h? ☐ Yes, ☐ No

3. Le participant a t-il réçu un antipaludique au cours des 14 derniers jours? ☐ Yes, ☐ No

   Si oui, spécifier le nom du médicament: --

3. Le participant a t-il migré hors de la zone d'étude au cours des 3 derniers mois? ☐ Yes, ☐ No

IV. Histoire Obstétricale ☐ NA

1. Etes vous déjà tombée enceinte? ☐ Yes, ☐ No

   Si oui, compléter le reste de la section ci-dessous:

2. Nombre total de grossesses (incluant la présente): |__|__|

3. Nombre total d'enfants vivants (parité): |__|__|

4. Nombre de grossesses > 28 semaines|__|__|: Naissance vivante |__|__| Fausse couche |__|__|

5. Nombre de grossesses < 28 semaines|__|__|: Avortement |__|__| interruption|__|__|

FORMULAIRE DE SELECTION ENROLEMENT

V. Histoire prénatale (de la présente grossesse): ☐ NA

1. Nombre de consultations pénatales (CPN) déjà réalisées: |__|__|

2. Date de la première CPN: |__|__|/|__|__|__|__|/|__|__|__|__| CSPS/CM: ______________________

3. Date de visite de la CPN la plus récente: |__|__|/|__|__|__|__|/|__|__|__|__|

4. Nombre de doses de TPI (Traitement Préventif Intermittent de la femme enceinte): |__|__|

5. Date de la dose de TPI la plus récente: |__|__|/|__|__|__|__|/|__|__|__|__|,

6. taux d'Hb (g/dl) le plus récent: |__|__| Date: |__|__|/|__|__|__|__|/|__|__|__|__|

7. Prise d'antipaludique au cours des deux dernières semaines: ☐ oui, ☐ Non

Si oui, Spécifier le médicament: ☐ AL, ☐ Autre……………………………………………….

et date du traitement: |__|__|/|__|__|__|__|/|__|__|__|__|,

8. Supplements: ☐ Non, ☐ Fer, ☐ Acide Folique, ☐ Vitamines

9. Nombre d'épisode de paludisme Durant cette grossesse: |__|__|

a. Date épisode 1: |__|__|/|__|__|__|__|/|__|__|__|__|,

Confirmé par TDR: ☐ Oui, ☐ Non

Prise d'antipaludique: ☐ AL, ☐ Autre……….. …….

b. Date episode 2: |__|__|/|__|__|__|__|/|__|__|__|__|, ☐ oui, ☐ Non

Confirmé par TDR: ☐ Oui, ☐ Non

Prise d'antipaludique: ☐ AL, ☐ Autre……….. …….

c. Date episode 3: |__|__|/|__|__|__|__|/|__|__|__|__|, ☐ oui, ☐ Non

Confirmé par TDR: ☐ Oui, ☐ Non

Prise d'antipaludique: ☐ AL, ☐ Autre……….. …….

Traitement antipaludique actuel : ☐ Yes, ☐ No

Si oui, spécifier la date: |__|__|/|__|__|__|__|/|__|__|__|__|,

10. Autres traitements récus pendant cette grossesse: ☐ NA

a. ……………………….. date début: |__|__|/|__|__|__|__|/|__|__|__|__|

date fin: |__|__|/|__|__|__|__|/|__|__|__|__| Raison: ……………………………….

b. ……………………….. date début: |__|__|/|__|__|__|__|/|__|__|__|__|

date fin: |__|__|/|__|__|__|__|/|__|__|__|__| Raison: ……………………………….

c. ……………………….. date début: |__|__|/|__|__|__|__|/|__|__|__|__|

date fin: |__|__|/|__|__|__|__|/|__|__|__|__| Raison: ……………………………….

11. Age estimatif de la grossesse:

Date des dernières règles |__|__|/|__|__|__|__|/|__|__|__|__|

Age gestationnel estimative à l'enrôlement: |__|__| Semaines

Hauteur utérine: |__|__|__| cm Estimé par: ……………………………………………………………………….

Biométrie foetale: ……

………

Note: ……

Date échographique estimative de l'accouchement |__|__|/|__|__|__|__|/|__|__|__|__|

VI. Examen Medical

Poids: |__|__|__|, |__| kg **taille**: |__|__|__|cm **IMC:** |__|__|, |__|Kg/m^2

Température: |__|__|, |__|°C **Presence de fièvre /d'histoire de fièvre:** ☐ Oui, ☐ Non

Tension Arterielle: |__|__|__|/|__|__|__|mmHg **Pouls:** |__|__|__|batt./min

VII. Critères d'inclusion et de non-inclusion: éligibilité pour l'étude

Critère d'inclusion		
**1.**	***La femme enceinte est-elle eligible pour l'enrôlement?***	☐ **NA**
	Sujet âgée ≥15-40 ans	☐ Oui, ☐ Non
	Sujet volontaire pour les prélèvements sanguins de cette étude?	☐ Oui, ☐ Non
	Sujet or RLA a t-il signé le consentement?	☐ Oui, ☐ Non
**2.**	***La femme non enceinte est-elle eligible pour l'enrôlement?***	☐ **NA**
	Sujet âgée ≥15-40 ans	☐ Oui, ☐ Non
	Sujet volontaire pour les prélèvements sanguins de cette étude?	☐ Oui, ☐ Non
	Sujet or RLA a t-il signé le consentement?	☐ Oui, ☐ Non
	Le test urinaire de grossesse est-il negative?	☐ Oui, ☐ Non
**3.**	***L'enfant d'âge scolaire est-il eligible pour l'enrôlement?***	☐ **NA**
	Sujet âgé ≥5-14 ans	☐ Oui, ☐ Non
	Sujet volontaire pour les prélèvements sanguins de cette étude?	☐ Oui, ☐ Non
	Sujet or RLA a t-il signé le consentement?	☐ Oui, ☐ Non
	Le test urinaire de grossesse est-il negative?	☐ Oui, ☐ Non
**Critères de non inclusion**		
	Paludisme compliqué (basé sur les critères OMS)	☐ Oui, ☐ Non
	Participation pécédente ou actuel à un essai de vaccin antipaludique	☐ Oui, ☐ Non
	Sujet sous chemioprophylaxie (exemple, sujets avec Drépanocytose)	☐ Oui, ☐ Non
	Transfusion sanguine récente (dans les 3 derniers mois)	☐ Oui, ☐ Non

Le participant est-il eligible pour l'étude? ☐ Oui, ☐ Non

Si oui, alors, poursuivre le reste du questionnaire.

Si non, arrêter le questionnaire ici, et référer le participant pour sa prise en charge s'il y a lieu.

VIII. Evaluations de Laboratoire

a. Le test urinaire de grossesse a t-il été réalisé? ☐ NA,

☐ Non, mais confirmé précédemment par ……………………………………………

☐ Oui, Si oui, préciser le résultat: **|__| 1**=Positif, **2**=Negatif, **3**=Invalide

b. Blood sampling

Blood sampling	Volume	Sampling performed
Goutte épaisse / frottis sanguin	20µL	☐ Oui, ☐ Non
RDT (if applicable: Fever or reported fever)	5µL	☐ Oui, ☐ Non
Hb	20µL	☐ Oui, ☐ Non
Sang dans un tampon RNA protect	200 µL (2 aliquots of 100 µL)	☐ Oui, ☐ Non
Taux sanguin de SP	100 µL	☐ Oui, ☐ Non
PfHRP-2 et autres marqueurs clinique	100 µL (50 – 60 µL de plasma + pellet)	☐ Oui, ☐ Non
Papier filtre pour l'analyse moléculaire des parasites et dosages immunologiques	60 µL	☐ Oui, ☐ Non

IX. Enrôlement du participant dans l'étude

Le sujet a t-il été enrôlé? ☐ Oui, ☐ Non

Si oui, Préciser son N° d'étude: **IMH**|__|__|__|- **P** ("-P" pour participante enceinte)

IMH|__|__|__|- **NP** ("-NP" pour participante Non enceinte)

IMH|__|__|__|- **C** ("-C" pour participant enfant)

Si femme enceinte:

La participante a t_elle été enrôlé : ☐ Primigravidae ☐ Secundigravidae ☐ Multigravidae?

Commentaire:

X. Facteurs de risque du paludisme

1. **Utilisation des moustiquaires** :

Dormez-vous sous habituellement sous une moustiquaire? ☐ Oui, ☐ Non

Avez-vous dormi sous une moustiquaire la nuit précedante? ☐ Oui, ☐ Non

Si non, donnez la raison: ………………………………………………………………

Si Oui, spécifier les conditions/l'état de la moustiquaire:

Age de la moustiquaire: |__|__| (mois),

Origine de la moustiquaire: ☐ PNLP, ☐ Commerciale

Nom de marque de la moustiquaire: ………………………………………………….

Intégrité de la moustiquaire: ☐ Entière, ☐ Trouée, ☐ Déchirée

Nombre de fois que la moustiquaire a été lavé |__|__|

2. **Utilisation d'autres mesures de protection antipaludique:**

Pulvérisation intradomiciliaire (PID) au cours des 3 derniers mois? ☐ Oui, ☐ Non

Spirale anti-moustique ☐ Oui, ☐ Non

Pulvérisation de bombe insecticide ☐ Oui, ☐ Non

Répulsifs traditionels ☐ Oui, ☐ Non

Répulsifs commerciaux ☐ Oui, ☐ Non

3. **Utilisation de medicaments antipalidiques**

Prise d'antipaludique au cours des deux dernières semaines: ☐ Oui, ☐ Non

Si oui, spécifier : ☐ AL, ☐ Autres, spécifier ……………………………………………

Date du traitement: |__|__|/|__|__|__|/|__|__|__|__|

Traitement antipaludique actuelle: ☐ Oui, ☐ Non

Si oui, spécifier ………………………………………………………………………………………………

Prise de Chimioprophylaxie du Paludisme Saisonier (CPS) ? ☐ Oui, ☐ Non

Si oui, date de la dernière dose? |__|__|/|__|__|__|/|__|__|__|__|

4. **Les habitudes de sommeil**

Heure estimative du coucher de la dernière nuit: |__|__|:|__|__|

Heure estimative du réveil (sortir du lit) ce matin: |__|__|:|__|__|

Combien de fois sortez-vous du lit habituellement au cours de la nuit? |__|__|

Combien de fois êtes-vous sorti du lit au cours de la nuit précédente? |__|__|

5. **Facteurs de risques socio-économiques:** ☐ NA (Si le participant est du groupe d'âge scolaire)

a). Groupe ethnique: ………………………………………….

b). Niveau d'éducation scolaire

☐ Non scolarisé ☐ Primaire ☐ Secondaire ☐ Universitaire

c). Profession:

☐ Cultivateur ☐ commerçante ☐ Fonctionnaire ☐ Auto-employeur

☐ Autre, spécifier…………………………………………………………………………………………………

d). **Possession de biens matériels:**

☐ Radio ☐ Téléphone mobile

e). Conditions du logement: Maison avec**:**

☐ Toiture en métal ☐ Toiture en paille ☐ Ouverture avant-toît

Présence de fénêtre: ☐ Oui ☐ Non, Si Oui, spécifier le nombre |__|__|

Position de la fénêtre avec la porte: ☐ Du côté de la porte et nombre|__|

☐ Derrière la porte et nombre|__|

Taille fénêtres: ☐ Petite ☐ Moyenne ☐ grande

Taille de la concession: Nombre de chambres |__|__|, Nombre de personnes |__|__|

Présence d'électricité: ☐ Oui, ☐ Non

f). Présence d'hôte alternatifs (animaux domestiques) : ☐ Oui, ☐ Non

g). Propriétaire terrien: ☐ Oui, ☐ Non

h). Distance au centre de santé: ☐ < 3km, ☐ 3-5 km, ☐ > 5km

j). Présence d'habits accrochés dans la chambre: ☐ Oui, ☐ Non

k). **Propréte de la maison:** ☐ Oui, ☐ Non

l). **Présence de déchets solides à proxilté de la concession:** ☐ Oui, ☐ Non

Date: |__|__|/|__|__|__|/|__|__|__|__| **Initiales and Signature de l'Investigateur**………………………..
